# Subcutaneous envafolimab monotherapy in patients with advanced defective mismatch repair/microsatellite instability high solid tumors

**DOI:** 10.1186/s13045-021-01095-1

**Published:** 2021-06-21

**Authors:** Jian Li, Yanhong Deng, Weijie Zhang, Ai-Ping Zhou, Weijian Guo, Jianwei Yang, Ying Yuan, Liangjun Zhu, Shukui Qin, Silong Xiang, Haolan Lu, John Gong, Ting Xu, David Liu, Lin Shen

**Affiliations:** 1 Department of Gastrointestinal Oncology, Key Laboratory of Carcinogenesis and Translational Research (Ministry of Education), Peking University Cancer Hospital and Institute, Beijing, China; 2 Sun Yat-Sen University, Guangzhou, China; 3 The First Affiliated Hospital of Zhengzhou University, Henan, China; 4 National Cancer Center/Cancer Hospital, Chinese Academy of Medical Sciences, Peking Union Medical College, Beijing, China; 5 Department of Medical Oncology, Fudan University Shanghai Cancer Center, Shanghai, China; 6 Department of Oncology, Shanghai Medical College, Fudan University, Shanghai, China; 7 Fujian Provincial Cancer Hospital, Fuzhou, China; 8 Department of Medical Oncology, Second Affiliated Hospital and Key Laboratory of Cancer Prevention and Intervention, China National Ministry of Education, Zhejiang University College of Medicine, Hangzhou, China; 9 Department of Internal Medicine, Jiangsu Cancer Hospital, Jiangsu Institute of Cancer Research, Affiliated Cancer Hospital of Nanjing Medical University, Nanjing, China; 10 PLA Cancer Center, Nanjing Bayi Hospital, Nanjing, China; 11 3D Medicines Co., Ltd, Sichuan, China; 12 Alphamab Co., Ltd, Suzhou, China

**Keywords:** Envafolimab, PD-L1, dMMR/MSI-H, Subcutaneous injection

## Abstract

**Background:**

Monoclonal antibodies targeting programmed death ligand 1 (PD-L1) signaling currently approved for defective mismatch repair (dMMR)/microsatellite instability high (MSI-H) tumors must be delivered by intravenous infusion. Envafolimab, a humanized single-domain anti-PD-L1 antibody fused to an Fc fragment, represents a potential advance because it can be conveniently administered subcutaneously.

**Methods:**

This open-label, single-arm, phase 2 study evaluated the efficacy and safety of envafolimab in patients with previously treated advanced dMMR/MSI-H tumors from 25 clinical sites across China. Adults with histologically confirmed locally advanced or metastatic malignant dMMR/MSI-H solid tumors received weekly 150 mg subcutaneous envafolimab injections in a 28-day treatment cycle. The primary efficacy endpoint was the objective response rate (assessed by a blinded independent review committee). Secondary efficacy outcomes were disease control rate, duration of response, progression-free survival, and overall survival.

**Results:**

One hundred and three patients (65 with colorectal cancer, 18 with gastric cancer, and 20 with other solid tumors) were enrolled. Median follow-up was 11.5 months. The objective response rate was 42.7% (95% confidence interval [CI] 33.0–52.8), and the disease control rate was 66.0% (95% CI 56.0–75.1). Median duration of response was not reached; the duration of response rate at 12 months was 92.2% (95% CI 77.5–97.4). Median progression-free survival was 11.1 months (95% CI 5.5 to not evaluable). Overall survival at 12 months was 74.6% (95% CI 64.7–82.1). Sixteen patients (16%) had at least one grade 3 or 4 related treatment-emergent adverse event. No grade 5 treatment-emergent adverse events related to envafolimab were reported. Injection site reactions, all grade 1–2, were reported in nine patients (9%), but there were no infusion reactions. Eight patients (8%) had grade 3 or 4 immune-related adverse events.

**Conclusions:**

This is the first pivotal phase 2 study to examine the efficacy and safety of a single-domain immune checkpoint antibody in the treatment of cancer. Envafolimab was effective and had acceptable safety in the treatment of previously treated advanced dMMR/MSI-H solid tumors. As the first single-domain PD-L1-targeting antibody administered by rapid subcutaneous injection, envafolimab has the potential to be a significant advance in the treatment of cancer.

*Trial registration* ClinicalTrials.gov, NCT03667170. Registered 10 September 2018—Retrospectively registered, https://clinicaltrials.gov/ct2/show/NCT03667170.

**Supplementary Information:**

The online version contains supplementary material available at 10.1186/s13045-021-01095-1.

## Background

Immune checkpoint inhibitors targeting programmed cell death protein 1 (PD-1) and programmed death ligand 1 (PD-L1) have emerged as attractive treatment options for various cancers. One of their applications is in the treatment of microsatellite instability high (MSI-H) tumors, in which microsatellite sequences accumulate mutations due to defective mismatch repair (dMMR). MSI-H tumors carry a high mutation burden, typically express cancer-specific neoantigens, and are sensitive to PD-1/PD-L1 blockade [1, 2]. The prevalence of dMMR/MSI-H varies according to cancer type. Cancers with the highest rates of MSI-H (> 10%) include colorectal cancer (CRC) and gastric cancer (GC) [2, 3].

In previously treated advanced dMMR/MSI-H cancers, improved treatments have been needed because of limited treatment options and poor prognosis [4–9]. PD-1/PD-L1-targeting monoclonal antibodies represent a significant advance in the treatment of these tumors. Two PD-1-targeting monoclonal antibodies, pembrolizumab and nivolumab, have been approved in the USA for the treatment of metastatic dMMR/MSI-H CRC in patients who have failed standard chemotherapy with fluoropyrimidine, oxaliplatin, and irinotecan [10, 11]. Pembrolizumab has also been approved as a first-line treatment for patients with advanced dMMR/MSI-H CRC and in patients with other dMMR/MSI-H solid tumor types who have failed at least one prior systemic treatment, and was the first drug granted a tumor-agonistic indication by the US Food & Drug Administration. In multicenter phase 2 clinical trials in patients with advanced dMMR/MSI-H cancers, pembrolizumab [12–14] and nivolumab [11, 15] have shown acceptable toxicity and promising efficacy, with objective response rates (ORRs) of 28–36%. However, pembrolizumab, nivolumab, and all other approved PD-1/PD-L1-targeting monoclonal antibodies are administered by intravenous infusion, which can be inconvenient for patients and increase utilization of medical resources, and in a minority of patients can cause life-threatening infusion reactions [10, 11].

Single-domain antibodies lacking the immunoglobulin light chain are a possible alternative to full monoclonal antibodies. Although they tend to have a short half-life due to rapid renal clearance, they are more soluble and stable than full monoclonal antibodies and, importantly, more rapidly penetrate tissues; collectively, these properties enable subcutaneous administration [16]. The first single-domain antibody received US Food & Drug Administration approval in 2019 [17, 18], but single-domain antibodies have not yet been approved for the treatment of cancer.

Envafolimab is a humanized camel-derived single-domain anti-PD-L1 antibody fused to a human immunoglobulin F_c_ fragment to prolong its half-life [19]. It has the potential to be a major advance in the treatment of cancer because it would be the first subcutaneous PD-1/PD-L1-targeting single-domain antibody for treating cancer. The full therapeutic dose of envafolimab (150 mg) can be delivered as a single 0.75-ml subcutaneous injection in under 30 s due to the high solubility of the fusion protein (200 mg/mL). Across phase 1 dose-escalation studies in advanced cancers, confirmed response rates ranged from 14 to 18%, and no infusion reactions and only two grade 3 treatment-related adverse events (AEs) were reported [20–22]. Pharmacokinetic analyses of envafolimab monotherapy showed that 150 mg once weekly would provide a sufficient exposure in cancer patients [23]. Here, we describe the results of a pivotal phase 2 study of the efficacy and safety of envafolimab in adults with advanced dMMR/MSI-H solid tumors.

## Methods

### Overall study design and ethics

This is a multicenter, open-label, single-arm phase 2 study (ClinicalTrials.gov number, NCT03667170) to evaluate the efficacy and safety of envafolimab in adults with locally advanced or metastatic dMMR/MSI-H malignant solid tumors who have failed at least one prior systemic therapy. Initiated in August 2018, the study is being conducted at 25 sites in China, where pembrolizumab and nivolumab are unavailable for the treatment of previously treated dMMR/MSI-H cancers. Enrolled patients received weekly subcutaneous injections of envafolimab 150 mg in a 28-day treatment cycle until disease progression, death, intolerable toxicity, or withdrawal of consent. Tumor imaging and assessment of objective response according to RECIST version 1.1 were performed every 8 weeks (± 7 days). A blinded independent review committee (BIRC) was established for central assessments, the results of which were not communicated to the investigators, who had sole responsibility for treatment decisions. Safety follow-up was performed 30 days after the last dose of envafolimab and survival follow-up every 12 weeks from disease progression or the last dose of envafolimab. A data monitoring committee reviewed the safety data periodically.

The study was approved at each site by the local independent ethics committee and was conducted in compliance with the Declaration of Helsinki, International Council for Harmonisation Good Clinical Practice guidelines, and applicable laws and regulations. All enrolled patients provided signed, informed consent for participation.

### Patients

Eligible patients were aged ≥ 18 years with histologically confirmed locally advanced or metastatic malignant solid tumors. They had MSI-H confirmed by PCR by a central laboratory (CRC and GC) or dMMR confirmed by immunohistochemistry by a local laboratory (other solid tumors), ≥ 1 measurable lesion (RECIST version 1.1), and failure of ≥ 1 prior systemic therapy. Patients also had to have adequate organ and bone marrow function, an expected survival of ≥ 12 weeks, and an Eastern Cooperative Oncology Group performance status of 0 or 1. Patients who had previously received immune checkpoint inhibitor therapy were excluded. Other exclusion criteria are listed in Additional file 1: Table S1.

### Outcomes

The primary efficacy endpoint was the confirmed ORR (complete or partial response) of envafolimab monotherapy according to RECIST version 1.1, as assessed by the BIRC. Secondary efficacy endpoints included investigator-assessed ORR, disease control rate (DCR; complete or partial response, or stable disease [≥ 6 weeks for BIRC assessments, no minimum duration for investigator assessments]), duration of response (DoR: time between the date of response and progression or death), progression-free survival (PFS: time between the first dose of envafolimab and progression or death) according to RECIST version 1.1, and overall survival (OS: time between the first dose of envafolimab and death).

Treatment-emergent adverse events (TEAEs) were collected from the first dose of envafolimab until the safety follow-up 30 days after the last dose. Serious adverse events (SAEs) occurring within 30 days after the last dose of envafolimab were followed up until 90 (± 7) days after the last dose of envafolimab or the initiation of other anti-tumor therapy. TEAEs were assessed for severity (graded according to NCI CTCAE version 4.03), relationship to envafolimab, seriousness, and action taken regarding envafolimab (dose interruption, discontinuation). Injection site reactions involving pain, reaction, discomfort, pruritus, swelling, and hypersensitivity were assessed as AEs of special interest.

Eleven categories of treatment-emergent immune-related adverse events (irAEs), identified using an algorithm developed with data from clinical studies of envafolimab monotherapy, were assessed based on guidelines from the Chinese Center for Drug Evaluation (Additional file 1: Table S2).

### Statistical analysis

Statistical analyses were performed using SAS 9.4 (SAS Institute Inc., Cary, NC).

Efficacy and safety analyses were conducted for the full analysis set, which comprised all enrolled patients who received ≥ 1 dose of envafolimab. The analyses included data collected up until June 19, 2020. The primary efficacy population (PEP) comprised patients with GC or with CRC previously treated with triplet therapy of fluorouracil, oxaliplatin, and irinotecan. Additional efficacy analyses were based on the overall study population, patients with CRC previously treated with fluorouracil plus oxaliplatin or fluorouracil plus irinotecan doublet therapy, all patients with CRC, and patients with solid tumors other than GC or CRC.

ORR and DCR were calculated based on best overall response. Two-sided 95% confidence intervals (CIs) were calculated using the Clopper–Pearson method. The protocol-specified success threshold for ORR was a lower 95% CI bound ≥ 15%. DoR, PFS, OS, and proportions of patients with DoR, PFS, and OS of at least 12 months were estimated by the Kaplan–Meier method. The corresponding 95% CIs were calculated using the Greenwood formula [24] with log–log transformation. For DoR and PFS, patients without disease progression or death were censored at the date of the last on-study tumor assessment. For OS, patients were censored at the date when they were last known to be alive. Safety outcomes were summarized using descriptive statistics.

## Results

### Patients

**Fig. 1:**
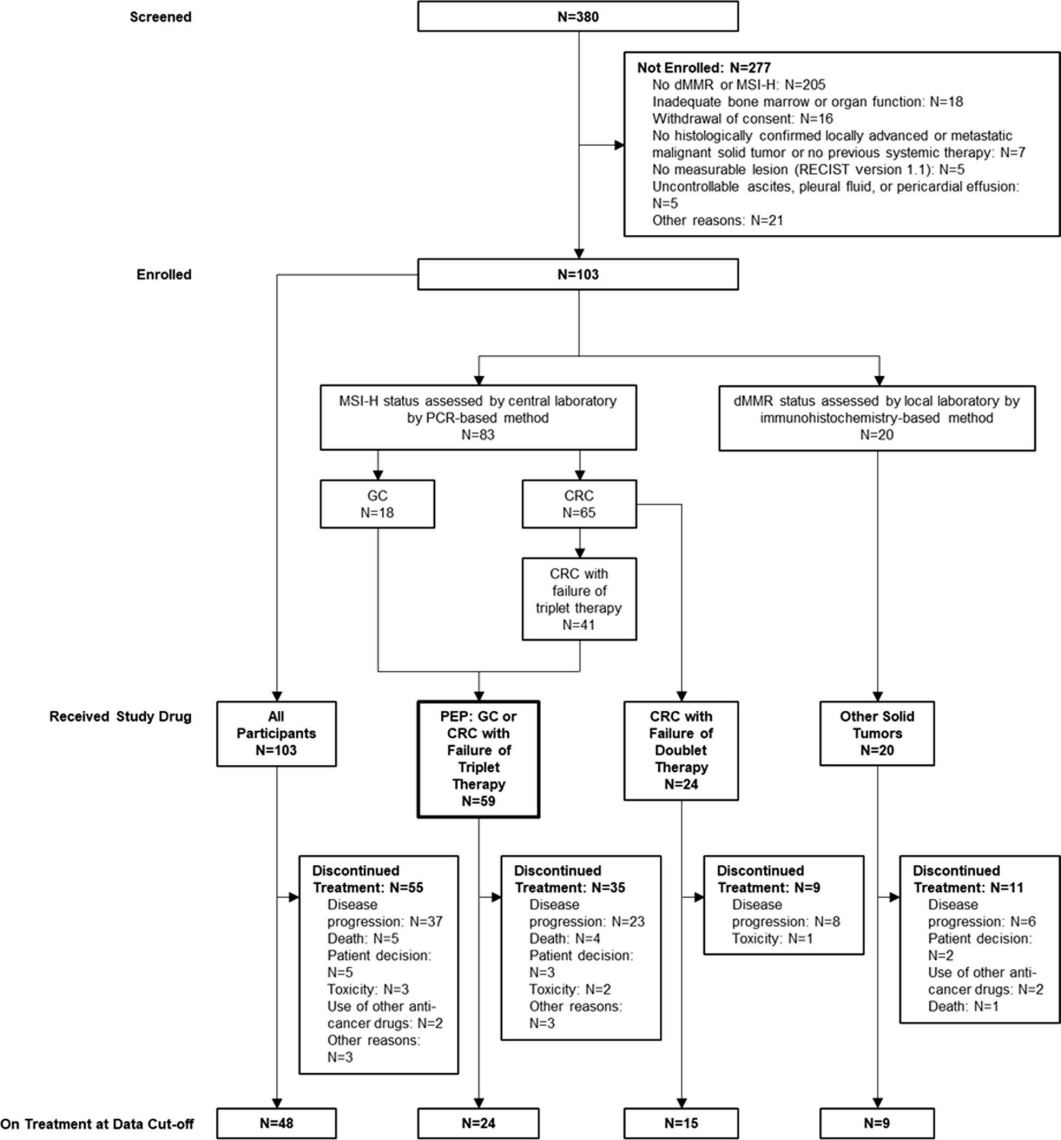
Patient disposition. CRC, colorectal cancer; dMMR, defective mismatch repair; GC, gastric cancer; MSI-H, microsatellite instability high; PEP, primary efficacy population

Between August 2018 and December 2019, the study enrolled 103 patients, all of whom received envafolimab (Fig. 1). For the overall population, median follow-up—defined as the median duration between the date of the first dose and the last date the patients were alive and followed for survival—was 11.5 months (the minimum follow-up for the last patient was 6.5 months). Fifty-five patients (53.4%) discontinued treatment with envafolimab, mostly due to disease progression. Three patients (2.9%) discontinued treatment because of toxicity of envafolimab.

In the overall population, median age was 53.0 years, and 63% of patients were male (Table 1). Most patients were Han Chinese (95%) and had a diagnosis of CRC (63%). Eighteen patients had GC. The most frequent types of other solid tumors were endometrial cancer (6 patients), hepatocellular cancer (2 patients), and hepatocholangiocarcinoma (2 patients). Eastern Cooperative Oncology Group performance status was 0 in 26% of patients and 1 in 74%. Participants had received a median of two prior systemic treatments (range 1–7).

**Table 1:** Baseline demographic and clinical characteristics

Characteristic	CRC			Non-CRC			Overall study population (*N* = 103)
	≥ 2 Prior therapies (*N* = 41)	1 Prior therapy (*N* = 24)	Total (*N* = 65)	GC (*N* = 18)	Other solid tumors (*N* = 20)	Total (*N* = 38)	
*Age (years)*							
Median	48.0	50.5	49.0	61.0	56.0	58.5	53.0
Range	25–77	22–76	22–77	34–72	36–75	34–75	22–77
*Sex, n (%)*							
Male	30 (73)	13 (54)	43 (66)	14 (78)	8 (40)	22 (58)	65 (63)
Female	11 (27)	11 (46)	22 (34)	4 (22)	12 (60)	16 (42)	38 (37)
*Ethnicity, n (%)*							
Han Chinese	39 (95)	21 (87.5)	60 (92)	18 (100)	20 (100)	38 (100)	98 (95)
Other	2 (5)	3 (12.5)	5 (8)	0	0	0	5 (5)
*ECOG PS, n (%)*							
0	10 (24)	7 (29)	17 (26)	3 (17)	7 (35)	10 (26)	27 (26)
1	31 (76)	17 (71)	48 (74)	15 (83)	13 (65)	28 (74)	76 (74)
*Cancer diagnosis, n (%)*							
Colorectal cancer	41	24	65	–	–	–	65 (63)
Gastric/gastroesophageal junction cancer	–	–	–	18	–	18	18 (17)
Other solid tumors	–	–	–	–	20	20	20 (19)
Endometrial cancer	–	–	–	–	6	6	6 (6)
Hepatocellular cancer	–	–	–	–	2	2	2 (2)
Hepatocholangiocarcinoma	–	–	–	–	2	2	2 (2)
Bladder cancer	–	–	–	–	1	1	1 (1)
Cervical cancer	–	–	–	–	1	1	1 (1)
Cholangiocarcinoma	–	–	–	–	1	1	1 (1)
Esophageal cancer	–	–	–	–	1	1	1 (1)
Non-small cell lung cancer	–	–	–	–	1	1	1 (1)
Osteosarcoma	–	–	–	–	1	1	1 (1)
Prostate cancer	–	–	–	–	1	1	1 (1)
Renal pelvic carcinoma	–	–	–	–	1	1	1 (1)
Urothelial carcinoma	–	–	–	–	1	1	1 (1)
Uterine sarcoma	–	–	–	–	1	1	1 (1)
*Number of prior systemic treatments*							
Median	3	2	3	2	2	2	2
Range	1–7	1–4	1–7	1–4	1–5	1–5	1–7

CRC with ≥ 2 prior therapies included patients previously treated with a fluoropyrimidine-, oxaliplatin-, and irinotecan-containing regimen

CRC with 1 prior therapy included patients previously treated with a fluoropyrimidine- and oxaliplatin- or fluoropyrimidine- and irinotecan-containing regimen

CRC, colorectal cancer; ECOG, Eastern Cooperative Oncology Group; GC, gastric cancer; PS, performance status

### Efficacy

BIRC-assessed confirmed ORR, the primary endpoint, was 42.7% (95% CI 33.0–52.8) for the overall study population (*N* = 103), 43.1% (95% CI 30.8–56.0) for all CRC patients (*N* = 65), 31.7% (95% CI 18.1–48.1) for CRC patients who had failed triplet therapy (*N* = 41), 62.5% (95% CI 40.6–81.2) for CRC patients who had failed doublet therapy (*N* = 24), 44.4% (95% CI 21.5–69.2) for GC (*N* = 18), and 40.0% (95% CI 19.1–63.9) for other solid tumors (*N* = 20) (Table 2). For the PEP (*N* = 59), the ORR was 35.6% (95% CI 23.6–49.1). Investigator-assessed ORR was 41.7% (95% CI 32.1–51.9) for the overall study population and 35.6% (95% CI 23.6–49.1) for the PEP.

**Table 2:** Efficacy outcomes

	CRC			Non-CRC			Overall study population (*N* = 103)
	≥ 2 Prior therapies (*N* = 41)	1 Prior therapy (*N* = 24)	Total (*N* = 65)	GC (*N* = 18)	Other solid tumors (*N* = 20)	Total (*N* = 38)	
*BIRC*							
ORR							
n (%)	13 (31.7)	15 (62.5)	28 (43.1)	8 (44.4)	8 (40.0)	16 (42.1)	44 (42.7)
95% CI	18.1–48.1	40.6–81.2	30.8–56.0	21.5–69.2	19.1–63.9	26.3–59.2	33.0–52.8
*DCR*							
n (%)	24 (58.5)	16 (66.7)	40 (61.5)	15 (83.3)	13 (65.0)	28 (73.7)	68 (66.0)
95% CI	42.1–73.7	44.7–84.4	48.6–73.3	58.6–96.4	40.8–84.6	56.9–86.6	56.0–75.1
*DoR*							
Range, months	1.05 + to 16.59 +	2.76 + to 14.98 +	1.87 to 14.98 +	1.05 + to 16.59 +	3.75 + to 14.72 +	1.05 + to 16.59 +	1.05 + to 16.59 +
≥ 12 months, % (95% CI)	74.6 (39.8–91.1)	100.0 (100.0–100.0)	88.4 (68.0–96.1)	100.0 (100.0–100.0)	100.0 (100.0–100.0)	100.0 (100.0–100.0)	92.2 (77.5–97.4)
*PFS*							
Median (95% CI), months	4.9 (1.9–9.9)	NR (1.8–NE)	7.2 (3.5–NE)	NR (11.1–NE)	NR (1.9–NE)	NR (5.5–NE)	11.1 (5.5–NE)
Range, months	0.85 to 16.76 +	0.76 to 18.27 +	0.76 to 18.27 +	1.41 to 18.43 +	0.03 + to 16.62 +	0.03 + to 18.43 +	0.03 + to 18.43 +
% at 12 months (95% CI)	32.1 (18.1–47.0)	62.5 (40.3–78.4)	43.7 (31.2–55.4)	58.0 (18.3–84.0)	52.6 (28.7–71.9)	57.7 (37.3–73.5)	48.5 (37.8–58.3)
*OS*							
Median (95% CI), months	NR (10.3–NE)	NR (NE–NE)	NR (NE–NE)	NR (NE–NE)	NR (5.9–NE)	NR (NE–NE)	NR (NE–NE)
% at 12 months (95% CI)	64.7 (47.7–77.4)	87.1 (65.2–95.7)	72.9 (60.1–82.2)	83.3 (56.8–94.3)	75.0 (50.0–88.7)	78.9 (62.1–88.8)	74.6 (64.7–82.1)

CRC with ≥ 2 prior therapies included patients previously treated with a fluoropyrimidine-, oxaliplatin-, and irinotecan-containing regimen

CRC with 1 prior therapy included patients previously treated with a fluoropyrimidine- and oxaliplatin- or fluoropyrimidine- and irinotecan-containing regimen

BIRC, blinded independent review committee; CI, confidence interval; CRC, colorectal cancer; DCR, disease control rate; DoR, duration of response; GC, gastric cancer; NE, not evaluable; NR, not reached; ORR, objective response rate; OS, overall survival; PFS, progression-free survival

*For ORR, DCR, and DoR, each complete response and partial response as the best overall response was confirmed 4 weeks later (per RECIST Version 1.1)

^+^Censored

BIRC-assessed DCR was 66.0% (95% CI 56.0–75.1) for the overall study population (Table 2) and 66.1% (95% CI 52.6–77.9) for the PEP. Investigator-assessed DCR was 72.8% (95% CI 63.2–81.1) for the overall study population and 74.6% (95% CI 61.6–85.0) for the PEP.

**Fig. 2:**
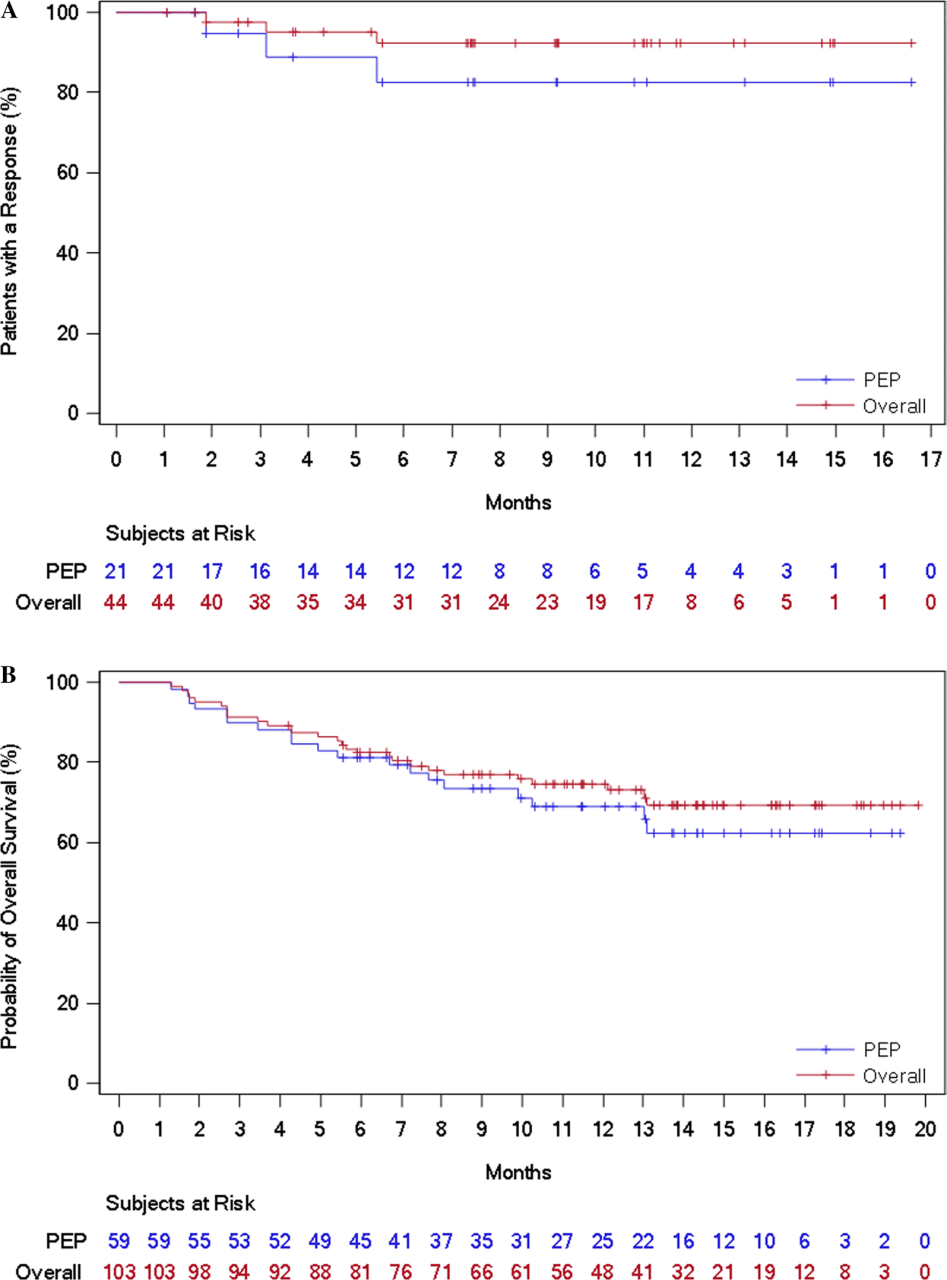
Time to event outcomes for the primary efficacy population and the overall study population. **a** Duration of response, assessed by the blinded independent review committee. **b** Overall survival

Median DoR was not reached for the overall study population or the other populations, including the PEP. The BIRC-assessed DoR rate at 12 months was 92.2% (95% CI 77.5–97.4) for the overall study population and 82.5% (95% CI 54.7–94.0) for the PEP (Table 2 and Fig. 2a). For patients with other solid tumors who achieved an objective response, the DoR rate at 12 months was 100.0% (Table 2 and Additional file 1: Figure S1).

BIRC-assessed median PFS was 11.1 months (95% CI 5.5 to not evaluable) for the overall study population and 7.5 months (95% CI 3.5 to not evaluable) for the PEP (Table 2 and Additional file 1: Figure S2). For both the overall study population and the PEP, median OS was not reached (Table 2 and Fig. 2b). For the overall study population, OS was 74.6% (95% CI 64.7–82.1) at 12 months.

### Safety

Ninety-nine patients (96%) had at least one TEAE, and 38 (37%) had at least one grade 3 or 4 TEAE (Table 3). Three grade 5 TEAEs were reported (lower gastrointestinal perforation, cholestatic jaundice, and death), none of which were considered to be related to envafolimab.

**Table 3:** Summary of adverse events (overall study population)

Outcome	Number of patients (%)		
		*N* = 103	
	All grades	Grade 3 or 4	Grade 5
TEAEs	99 (96)	38 (37)	3 (3)
Study drug-related TEAEs	87 (84)	16 (16)	0
*Related TEAEs with rate* ≥ *10%*			
Decreased white blood cell count	17 (17)	0	0
Asthenia	17 (17)	0	0
Rash	16 (16)	1 (1)	0
Hypothyroidism	16 (16)	0	0
Hyperthyroidism	12 (12)	0	0
Decreased neutrophil count	12 (12)	1 (1)	0
Anemia	12 (12)	5 (5)	0
SAEs	27 (26)	20 (19)	3 (3)
Related SAEs	10 (10)	9 (9)	0
Related TEAEs leading to discontinuation	3 (3)	3 (3)	0
irAEs	44 (43)	8 (8)	0
irAEs leading to discontinuation	3 (3)	3 (3)	0
*Injection site reactions*	9 (9)	0	0
Injection site pain	2 (2)	0	0
Injection site reaction	6 (6)	0	0
Injection site swelling	1 (1)	0	0

AE, adverse event; irAE, immune-related adverse event; SAE, serious adverse event; TEAE, treatment-emergent adverse event

Eighty-seven patients (84%) had at least one envafolimab-related TEAE, and 16 (16%) had at least one grade 3 or 4 related TEAE. The most frequent related TEAEs (≥ 10% of patients) were decreased white blood cell count (17%), asthenia (17%), rash (16%), hypothyroidism (16%), hyperthyroidism (12%), decreased neutrophil count (12%), and anemia (12%) (Table 3). Related TEAEs leading to treatment interruption occurred in 20 patients (19%).

Twenty-seven patients (26%) had at least one SAE, and 10 (10%) had at least one related SAE. The related SAEs were anemia (*n* = 3), abnormal hepatic function (*n* = 2), and hepatitis, immune-mediated hepatitis, diarrhea, esophageal obstruction, gastric hemorrhage, decreased appetite, decreased platelet count, myocarditis, and nephritis (*n* = 1 each).

Forty-four patients (43%) had treatment-emergent irAEs (Additional file 1: Table S3). Eight patients (8%) had grade 3 or 4 irAEs. The most frequent irAEs were endocrine disorders, with 23 patients (22%) experiencing hypothyroidism-related AEs and 21 (20%) hyperthyroidism-related AEs. Seven patients (7%) experienced immune-related adverse skin reactions, including rash (*n* = 4) and allergic dermatitis, eczema, and urticaria (*n* = 1 each). Grade 3 immune-related hepatitis occurred in four patients (4%). Treatment was permanently discontinued in three of these patients, one of whom also had grade 2 immune-related myocarditis. One patient experienced grade 3 immune-related diarrhea, and another patient experienced grade 3 immune-related pancreatitis (asymptomatic amylase increase). No immune-related pneumonitis, immune-related colitis, immune-related nephritis, immune-related thrombocytopenia, or immune-related neurological AEs were reported.

Nine patients (9%) had injection site reactions, all of which were grade 1 or 2 (Table 3). No infusion-related reactions were reported.

## Discussion

The current study was the first pivotal phase 2 trial to examine the efficacy and safety of a single-domain antibody in the treatment of cancer [18]. This study showed that envafolimab, a single-domain anti-PD-L1 antibody fusion protein administered by subcutaneous injection, was effective and had acceptable safety for the treatment of previously treated advanced dMMR/MSI-H solid tumors. Efficacy of envafolimab was similar to that reported in phase 2 trials for the intravenously administered monoclonal antibodies pembrolizumab and nivolumab in previously treated metastatic dMMR/MSI-H CRC [13, 15] and for pembrolizumab in previously treated dMMR/MSI-H non-CRC [14]. In addition to demonstrating the safety and efficacy of a subcutaneously administered single-domain antibody for treating cancer, the current study is the first to provide evidence that a single-domain anti-PD-L1 antibody has similar efficacy as full anti-PD-1 monoclonal antibodies in treating dMMR/MSI-H cancer.

Overall rates of grade 3 and 4 irAEs and drug-related AEs were in line with expectations for a monoclonal antibody targeting PD-L1 signaling [13, 15]. The most commonly reported drug-related TEAEs included decreased white blood cell count, asthenia, rash, hypothyroidism, and decreased neutrophil count, which have been reported for other PD-1/PD-L1 inhibitors [13, 15, 25, 26]. There were no infusion reactions, and injection site reactions were infrequent and of mild or moderate severity. Importantly, no cases of immune-related pneumonitis or colitis were reported in this study as of the data cutoff.

Since their initial approval in 2014, PD-1/PD-L1-targeting monoclonal antibodies have rapidly become a pillar of cancer treatment across many tumor types. However, because they are administered by intravenous infusion, infusion reactions can occur. Subcutaneous injection of envafolimab avoids the infusion reactions, though infrequent, seen with pembrolizumab and nivolumab [10, 11]. Also, envafolimab takes less than 30 s to administer, compared with 30 min or longer for intravenous infusion of currently available PD-1/PD-L1-targeting monoclonal antibodies. These represent significant advantages for patients and medical staff. They are expected to improve patient compliance and reduce medical resource utilization and are pertinent during the ongoing pandemic.

These advantages of envafolimab are expected to grow, because PD-1/PD-L1-targeting monoclonal antibodies are moving into first-line and adjuvant/neoadjuvant treatment across tumor types where chronic dosing is common. It is therefore noteworthy that, for patients with CRC in the present study, efficacy outcomes (including ORR and PFS) tended to be better for those who had failed one prior line of therapy than for those who had failed at least two prior lines of therapy. This suggests that envafolimab may be more effective in earlier-line treatment of advanced dMMR/MSI-H CRC, as reported for pembrolizumab [13, 27]. However, these observations should be interpreted with caution due to the limited numbers of patients.

The ORR and DoR were generally similar across tumor types, despite the different test methods applied (local versus central), an observation also made for pembrolizumab [28] and nivolumab [29]. Time to response and temporal response patterns for envafolimab were also consistent with pembrolizumab and nivolumab in dMMR/MSI-H solid tumors [13–15]. The results of this study support dMMR/MSI-H status as a valid tumor-agnostic biomarker for PD-L1 inhibitors. However, limitations of the study include the single-arm design, small sample for tumor types other than CRC and GC, and potential negative impact on efficacy due to possible heterogeneity of MSI-H status not detected by available tests.

## Conclusions

As the first single-domain PD-L1-targeting antibody administered by rapid subcutaneous injection, envafolimab has the potential to be a significant advance in the treatment of cancer. To confirm the current findings and generate more data for underrepresented tumor types in a larger population, the study is being expanded to become a confirmatory trial enrolling at least 200 patients with advanced dMMR/MSI-H solid tumors. Envafolimab is also being developed in other cancer indications where PD-1/PD-L1-targeting monoclonal antibodies have been shown to be effective.

## Supplementary Information

**Additional file 1**. **Table S1.** Exclusion criteria. **Table S2.** Algorithm for identifying immune-related adverse events. **Table S3.** Summary of immune-related adverse events. **Figure S1.** Duration of response, assessed by the blinded independent review committee, for the population with other solid tumors. **Figure S2.** Kaplan-Meier curves showing progression-free survival, assessed by the blinded independent review committee, for the overall study population and the primary efficacy population (PEP).

## Data Availability

The datasets used and/or analyzed during the current study are available from the corresponding author on reasonable request.

## Abbreviations

PD-1: Programmed cell death protein 1
PD-L1: Programmed death ligand 1
MSI-H: Microsatellite instability high
dMMR: Defective mismatch repair
CRC: Colorectal cancer
GC: Gastric cancer
AE: Adverse event
BIRC: Blinded independent review committee
ORR: Objective response rate
DCR: Disease control rate
DoR: Duration of response
PFS: Progression-free survival
OS: Overall survival
TEAE: Treatment-emergent adverse event
SAE: Serious adverse event
irAE: Immune-related adverse event
PEP: Primary efficacy population
CI: Confidence interval

## References

[1] Le DT, Uram JN, Wang H, Bartlett BR, Kemberling H, Eyring AD (2015). PD-1 blockade in tumors with mismatch-repair deficiency. N Engl J Med.

[2] Bonneville R, Krook MA, Kautto EA, Miya J, Wing MR, Chen HZ, et al. Landscape of microsatellite instability across 39 cancer types. JCO Precis Oncol. 2017;2017. 10.1200/PO.17.00073 PMC5972025 29850653

[3] Amonkar M, Lorenzi M, Zhang J, Mehta S, Liaw K-L. Structured literature review (SLR) and meta-analyses of the prevalence of microsatellite instability high (MSI-H) and deficient mismatch repair (dMMR) in gastric, colorectal, and esophageal cancers. J Clin Oncol. 2019;37.

[4] Koopman M, Kortman GA, Mekenkamp L, Ligtenberg MJ, Hoogerbrugge N, Antonini NF (2009). Deficient mismatch repair system in patients with sporadic advanced colorectal cancer. Br J Cancer.

[5] Tran B, Kopetz S, Tie J, Gibbs P, Jiang ZQ, Lieu CH (2011). Impact of BRAF mutation and microsatellite instability on the pattern of metastatic spread and prognosis in metastatic colorectal cancer. Cancer.

[6] Venderbosch S, Nagtegaal ID, Maughan TS, Smith CG, Cheadle JP, Fisher D (2014). Mismatch repair status and BRAF mutation status in metastatic colorectal cancer patients: a pooled analysis of the CAIRO, CAIRO2, COIN, and FOCUS studies. Clin Cancer Res.

[7] Kim CG, Ahn JB, Jung M, Beom SH, Kim C, Kim JH (2016). Effects of microsatellite instability on recurrence patterns and outcomes in colorectal cancers. Br J Cancer.

[8] Alex AK, Siqueira S, Coudry R, Santos J, Alves M, Hoff PM (2017). Response to chemotherapy and prognosis in metastatic colorectal cancer with DNA deficient mismatch repair. Clin Colorectal Cancer.

[9] Janjigian YY, Sanchez-Vega F, Jonsson P, Chatila WK, Hechtman JF, Ku GY (2018). Genetic predictors of response to systemic therapy in esophagogastric cancer. Cancer Discov.

[10] KEYTRUDA® (pembrolizumab). Full prescribing information. Merck Sharp & Dohme Corp. 2020. https://www.accessdata.fda.gov/drugsatfda_docs/label/2019/125514s040lbl.pdf. Accessed 25 May 2020.

[11] OPDIVO (nivolumab). Full prescribing information. Bristol-Myers Squibb Company. 2019. https://www.accessdata.fda.gov/drugsatfda_docs/label/2019/125554s070lbl.pdf. Accessed 25 May 2020.

[12] Diaz L, Marabelle A, Kim TW, Geva R, Van Cutsem E, André T (2017). Efficacy of pembrolizumab in phase 2 KEYNOTE-164 and KEYNOTE-158 studies of microsatellite instability high cancers. Ann Oncol.

[13] Le DT, Kim TW, Van Cutsem E, Geva R, Jager D, Hara H (2020). Phase II open-label study of pembrolizumab in treatment-refractory, microsatellite Instability-high/mismatch repair-deficient metastatic colorectal cancer: KEYNOTE-164. J Clin Oncol.

[14] Marabelle A, Le DT, Ascierto PA, Di Giacomo AM, De Jesus-Acosta A, Delord JP (2020). Efficacy of pembrolizumab in patients with noncolorectal high microsatellite instability/mismatch repair-deficient cancer: results from the phase II KEYNOTE-158 study. J Clin Oncol.

[15] Overman MJ, McDermott R, Leach JL, Lonardi S, Lenz HJ, Morse MA (2017). Nivolumab in patients with metastatic DNA mismatch repair-deficient or microsatellite instability-high colorectal cancer (CheckMate 142): an open-label, multicentre, phase 2 study. Lancet Oncol.

[16] Harmsen MM, De Haard HJ (2007). Properties, production, and applications of camelid single-domain antibody fragments. Appl Microbiol Biotechnol.

[17] Scully M, Cataland SR, Peyvandi F, Coppo P, Knobl P, Kremer Hovinga JA (2019). Caplacizumab treatment for acquired thrombotic thrombocytopenic purpura. N Engl J Med.

[18] Morrison C (2019). Nanobody approval gives domain antibodies a boost. Nat Rev Drug Discov.

[19] Zhang F, Wei H, Wang X, Bai Y, Wang P, Wu J (2017). Structural basis of a novel PD-L1 nanobody for immune checkpoint blockade. Cell Discov.

[20] Papadopoulos KP, Harb W, Lu N, Ma X, He Y, Yuan L (2018). Phase I study of KN035, a novel fusion anti-PD-L1 antibody administered subcutaneously in patients with advanced solid tumors in the USA. Ann Oncol.

[21] Xu J-M, Qin S, Zhang Y, Zhang Y, Jia R, Liu R (2019). Phase I study of KN035, the first subcutaneously administered, novel fusion anti-PD-L1 antibody in patients with advanced solid tumors in China. J Clin Oncol.

[22] Shimizu T, Eguchi Nakajima T, Lu N, Xue S, Xu W, Fu M (2019). Phase I safety and pharmacokinetic study of KN035, the first subcutaneously administered, novel fusion anti-PD-L1 antibody in Japanese patients with advanced solid tumors. J Clin Oncol.

[23] Papadopoulos KP, Harb W, Peer CJ, Hua Q, Xu S, Lu H, Lu N, He Y, Xu T, Dong R, Gong J, Liu D. First-in-Human Phase I Study of Envafolimab, a novel subcutaneous single-domain Anti-PD-L1 antibody, in patients with advanced solid tumors. Oncologist. 2021. 10.1002/onco.13817. 10.1002/onco.13817 PMC8417852 33973293

[24] Kalbfleisch JD, Prentice RL (1980). The statistical analysis of failure time data.

[25] Tang B, Yan X, Sheng X, Si L, Cui C, Kong Y (2019). Safety and clinical activity with an anti-PD-1 antibody JS001 in advanced melanoma or urologic cancer patients. J Hematol Oncol.

[26] Shi Y, Su H, Song Y, Jiang W, Sun X, Qian W (2019). Safety and activity of sintilimab in patients with relapsed or refractory classical Hodgkin lymphoma (ORIENT-1): a multicentre, single-arm, phase 2 trial. Lancet Haematol.

[27] Andre T, Shiu K-K, Kim TW, Vittrup Jensen B, Jensen LH, Punt CJA, et al. First-line therapy of pembrolizumab versus standard of care (SOC) in microsatellite instability-high/mismatch repair deficient metastatic colorectal cancer: the phase III, KEYNOTE-177 study. American Society of Clinical Oncology (ASCO) 2020 Annual Meeting; 2020; Alexandria.

[28] FDA grants accelerated approval to pembrolizumab for first tissue/site agnostic indication (press release). U.S. Food and Drug Administration, 2017.

[29] Kopetz S, Lonardi S, McDermott RS, Aglietta M, Hendlisz A, Morse M (2017). Concordance of DNA mismatch repair deficient (dMMR)/microsatellite instability (MSI) assessment by local and central testing in patients with metastatic CRC (mCRC) receiving nivolumab (nivo) in CheckMate 142 study. J Clin Oncol.

