# Supplementary material for: Subcutaneous envafolimab monotherapy in patients with advanced defective mismatch repair/microsatellite instability high solid tumors
Source: J Hematol Oncol. 2021 Jun 21;14:95. doi: 10.1186/s13045-021-01095-1 (PMC8218452; doi:10.1186/s13045-021-01095-1)
Supplement: Supplementary file 1 — Additional file 1. Table S1. Exclusion criteria. Table S2. Algorithm for identifying immune-related adverse events. Table S3. Summary of immune-related adverse events. Figure S1. Duration of response, assessed by the blinded independent review committee, for the population with other solid tumors. Figure S2. Kaplan-Meier curves showing progression-free survival, assessed by the blinded independent review committee, for the overall study population and the primary efficacy population (PEP). [file 13045_2021_1095_MOESM1_ESM.docx]

**Subcutaneous Envafolimab Monotherapy in Patients with Advanced Defective Mismatch Repair/Microsatellite Instability High** **Solid Tumors**

Jian Li, MD, Yanhong Deng, MD, Weijie Zhang, MD, Ai-Ping Zhou, MD, Weijian Guo, MD, Jianwei Yang, MD, Ying Yuan, MD, Liangjun Zhu, MD, Shukui Qin, MD, Silong Xiang, MS, Haolan Lu, PhD, John Gong, MD, PhD, Ting Xu, PhD, David Liu, MD, PhD, Lin Shen, MD

# Additional file 1

# Supplementary Tables and Figures

**Element**

Additional file 1: Table S1: Exclusion criteria

Additional file 1: Table S2: Algorithm for identifying immune-related adverse events

Additional file 1: Table S3: Summary of immune-related adverse events

Additional file 1: Figure S1. Duration of response, assessed by the blinded independent review committee, for the population with other solid tumors

Additional file 1: Figure S2. Kaplan-Meier curves showing progression-free survival, assessed by the blinded independent review committee, for the overall study population and the primary efficacy population (PEP)

**Page**

2

3

4

5

6

**Additional file 1: Table S1. Exclusion criteria**

| **Applicable to** | **Exclusion criterion** |
| --- | --- |
| All patients | Participation in a clinical trial of another study drug or investigational device within 28 days before the first dose of study drug treatment or anti-tumor therapy within 2 weeks before the first dose of study drug |
|  | Failure of toxicities due to prior anti-tumor treatment to recover to grade 0 or 1 (alopecia and grade ≤2 peripheral neurotoxicity due to chemotherapy were allowed) |
|  | Prior immune checkpoint inhibitor therapy |
|  | Major surgery (except biopsy) within 4 weeks before the first dose of study drug or incomplete healing of the surgical incision |
|  | Ascites requiring drainage or diuretic treatment, or pleural or pericardial effusion requiring drainage with/without symptoms of tachypnea within 2 weeks before the first dose of study drug |
|  | Symptomatic brain metastases or spinal cord compression |
|  | Another malignancy in the previous 5 years, except for basal cell or squamous cell carcinoma after radical surgery or carcinoma *in situ* |
|  | Known or suspected active autoimmune disorder |
|  | History of HIV or active bacterial or fungal infection requiring systemic treatment within 14 days before the first dose of study drug |
|  | History of interstitial lung disease, drug-induced interstitial lung disease, radiation pneumonitis, symptomatic interstitial lung disease, or any evidence of active pneumonia on chest CT scan within 4 weeks before the first dose of study drug |
|  | Hepatitis B virus DNA ≥10^4^ copies/mL in serum testing at screening |
|  | History of clinically significant cardiovascular disease |
|  | Inadequately controlled thyroid dysfunction |
|  | Clinically significant serum electrolyte abnormality |
|  | Use of immunosuppressive drugs within 2 weeks before the first dose of study drug, excluding local glucocorticoids or systemic glucocorticoids at a dose not exceeding 10 mg/day |
|  | Vaccination with a live vaccine within 4 weeks before the first dose of study drug or planned vaccination with a live vaccination during the study |
|  | History of severe allergic reaction to chimeric or humanized antibodies or fusion proteins, or known allergy to biological products produced using Chinese hamster ovary cells or to any component of the study drug |
|  | Disease, metabolic disorder, or laboratory abnormality that, in the opinion of the investigator, could compromise patient safety or interpretation of the study results if they were to be included in the study |
| Females of childbearing potential | Current pregnancy or lactation |
|  | Unwillingness to use an effective method of contraception |

CT, computed tomography; DNA, deoxyribonucleic acid; HIV, human immunodeficiency virus.

**Additional file 1: Table S2. Algorithm for identifying immune-related adverse events**

| **Step 1** | Summarize the preferred terms of AEs occurring in all clinical studies of envafolimab monotherapy^#^ |
| --- | --- |
| **Step 2** | Medically identify all potential irAE preferred terms and categorize them into 11 categories of irAEs* (refer to the approved irAE listing for anti-PD-1 antibodies published by the Chinese Center for Drug Evaluation) |
| **Step 3** | Systematically screen the preferred terms in Step 2 according to the following inclusion/exclusion rules: |
|  | 1. For grade 3 or higher AEs, exclude those considered by investigators as “not related” |
|  | 1. For grade 1-2 AEs, exclude the following:   - AEs considered by the investigator as “not related" or “doubtfully related”  - AEs with “no treatment with systemic hormones,” with the following exceptions:  - For skin AEs, exclude AEs with “no treatment with topical or systemic hormones”  - For endocrine and pneumonitis AEs, include all AEs regardless of medicinal treatment |
| **Step 4** | Medically review all AEs from Step 3 by applying the following exclusion rule:  - Exclude AEs with unequivocal nonimmune etiology (e.g. concomitant medications, previous medical history, tumor progression, infections) |
| **Step 5** | Complete the final listing of irAEs according to the 11 categories of irAEs |

Note: the analysis of irAEs was based on treatment-emergent adverse events.

^#^The data cut-off date for studies of envafolimab monotherapy was October 14, 2019. Two preferred terms, “immune-mediated hepatitis” and “amylase increased,” were added following a subsequent review of data from the present study.

*Immune-related pneumonitis, immune-related diarrhea and colitis, immune-related hepatitis, immune-related nephritis, immune-related endocrine disorders (hypothyroidism, hyperthyroidism, thyroiditis, hypophysitis, adrenocortical insufficiency, hyperglycemia, and type 1 diabetes mellitus), immune-related skin adverse reactions, immune-related pancreatitis, immune-related thrombocytopenia, immune-related myocarditis, immune-related neurologic adverse events, and other immune-related adverse events.

AE, adverse event; irAE, immune-related adverse event; PD, programmed death.

**Additional file 1: Table S3. Summary of immune-related adverse events**

| **Outcome** | **Number of patients (%)** | | |
| --- | --- | --- | --- |
|  | **N=103** | | |
|  | **All grades** | **Grade 3 or 4** | **Grade 5** |
| Treatment-emergent immune-related AEs | 44 (42.7) | 8 (7.8) | 0 |
| Immune-related endocrine disorders | 35 (34.0) | 0 | 0 |
| Hypothyroidism-related AEs ^a^ | 23 (22.3) | 0 | 0 |
| Median time to onset (weeks) | 16.0 |  |  |
| Range | 4.0-47.9 |  |  |
| Leading to dose interruption | 1 (1.0) |  |  |
| Leading to permanent discontinuation | - |  |  |
| Hyperthyroidism-related AEs ^b^ | 21 (20.4) | 0 | 0 |
| Median time to onset (weeks) | 8.1 |  |  |
| Range | 3.7-68.1 |  |  |
| Leading to dose interruption | - |  |  |
| Leading to permanent discontinuation | - |  |  |
| Hyperglycemia | 1 (1.0) | 0 | 0 |
| Immune-related adverse skin reactions | 7 (6.8) | 2 (1.9) | 0 |
| Median time to onset(weeks) | 2.9 |  |  |
| Range | 0.7-37.6 |  |  |
| Leading to dose interruption | 1 (1.0) |  |  |
| Leading to permanent discontinuation | - |  |  |
| Rash | 4 (3.9) | 1 (1.0) | 0 |
| Immune-related hepatitis | 4 (3.9) | 4 (3.9) | 0 |
| Median time to onset (weeks) | 34.6 |  |  |
| Range | 2.0-56.6 |  |  |
| Leading to dose interruption | 1 (1.0) |  |  |
| Leading to permanent discontinuation | 3 (2.9) |  |  |
| Immune-related diarrhea and colitis | 1 (1.0) | 1 (1.0) | 0 |
| Diarrhea | 1 (1.0) | 1 (1.0) | 0 |
| Immune-related myocarditis | 1 (1.0) | 0 | 0 |
| Myocarditis | 1 (1.0) | 0 | 0 |
| Leading to dose interruption | - |  |  |
| Leading to permanent discontinuation | 1 (1.0) |  |  |
| Corticosteroid administration | 1 (1.0) |  |  |
| Immune-related pancreatitis | 1 (1.0) | 1 (1.0) | 0 |
| Increased amylase | 1 (1.0) | 1 (1.0) | 0 |

^a^ Hypothyroidism, blood-thyroid stimulating hormone increased, free thyroxine decreased, and free triiodothyronine decreased.

^b^ Hyperthyroidism, free triiodothyronine increased, and blood thyroid-stimulating hormone decreased.

AE, adverse event.

**Additional file 1: Figure S1. Duration of response, assessed by the blinded independent review committee, for the population with other solid tumors.** +, censored.

**Additional file 1: Figure S2. Kaplan-Meier curves showing progression-free survival, assessed by the blinded independent review committee, for the overall study population and the primary efficacy population (PEP).**

+, censored.
